# Supplementary material for: Ursolic Acid Alleviates Cancer Cachexia and Prevents Muscle Wasting via Activating SIRT1
Source: Cancers (Basel). 2023 Apr 20;15(8):2378. doi: 10.3390/cancers15082378 (PMC10136986; doi:10.3390/cancers15082378)

## Figure 1F

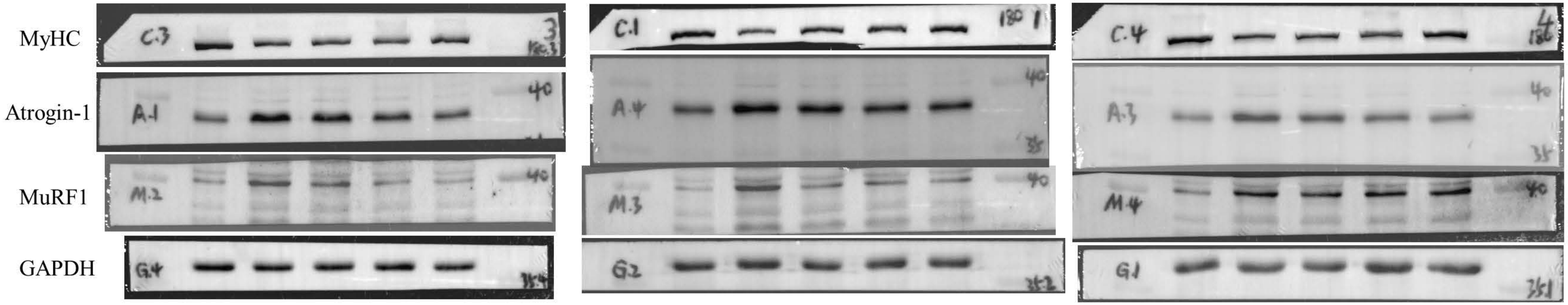


## Figure 2I

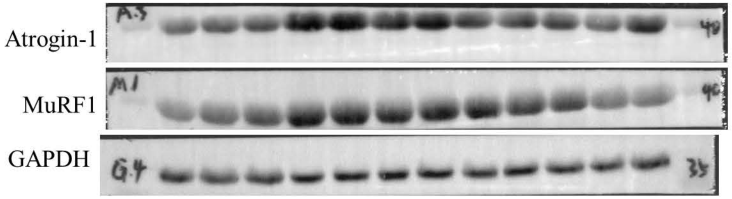


## Figure 3C

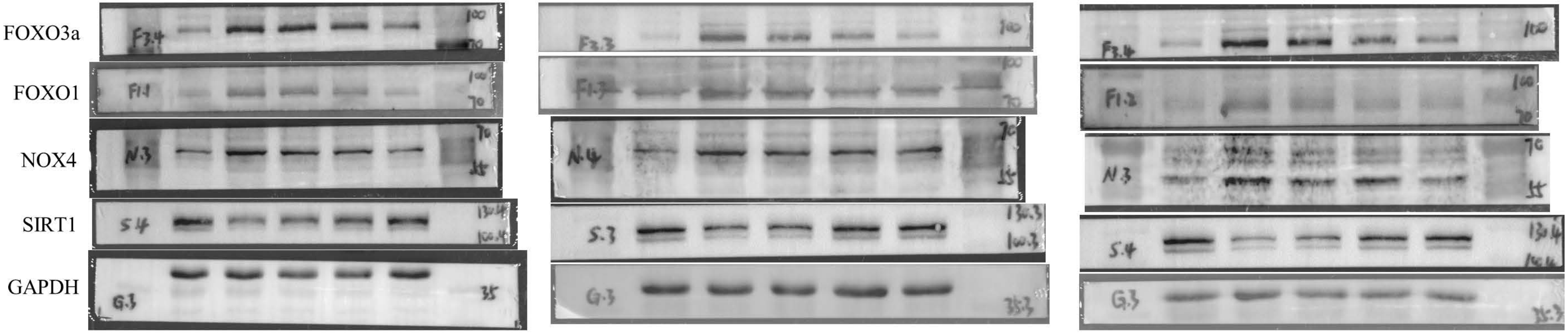

Figure 3D

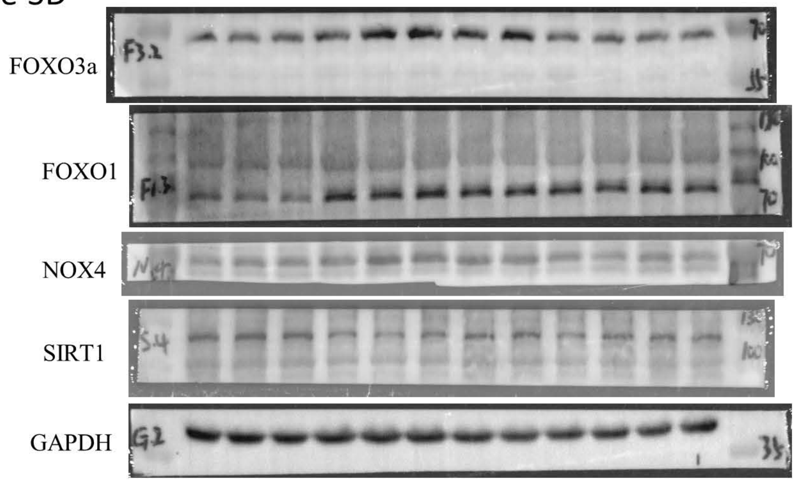


Figure 4B

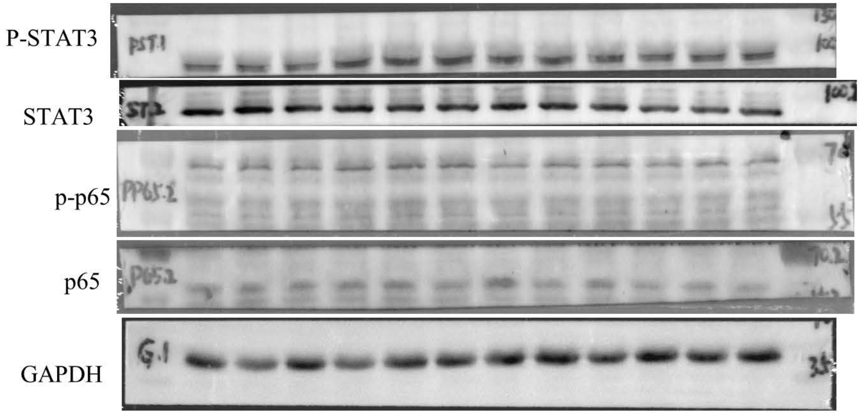


Figure 4A

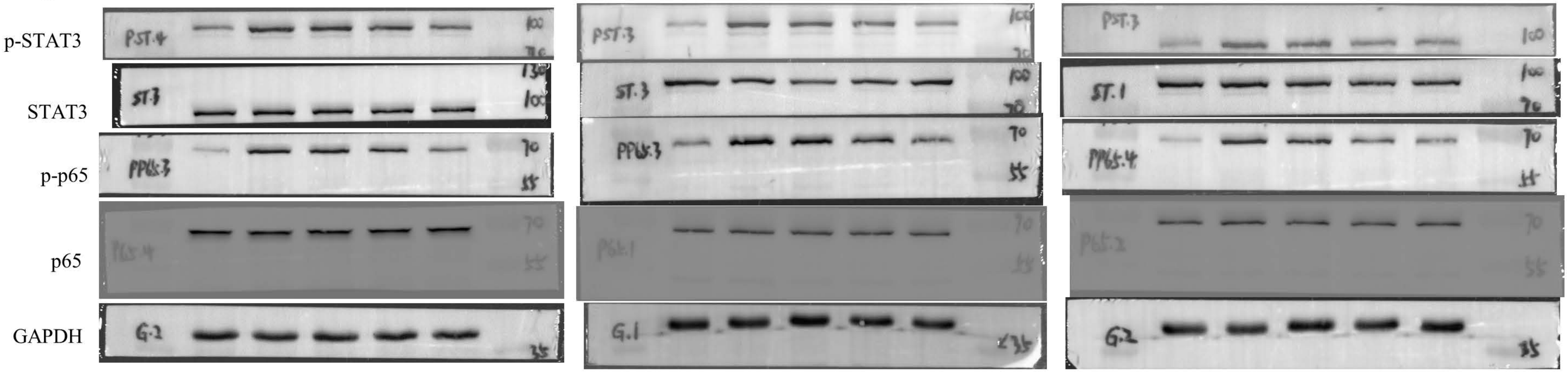


Figure 5D

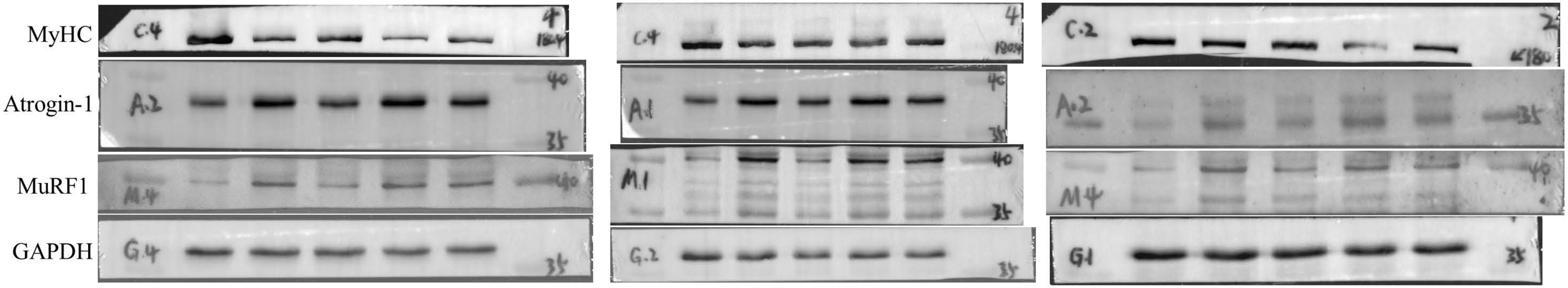

Figure 5E

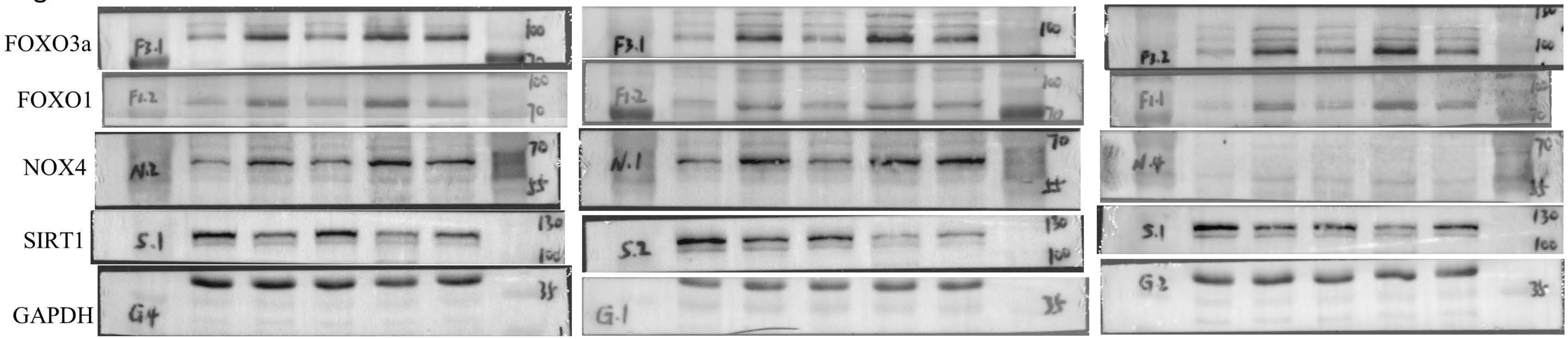


Figure 5F

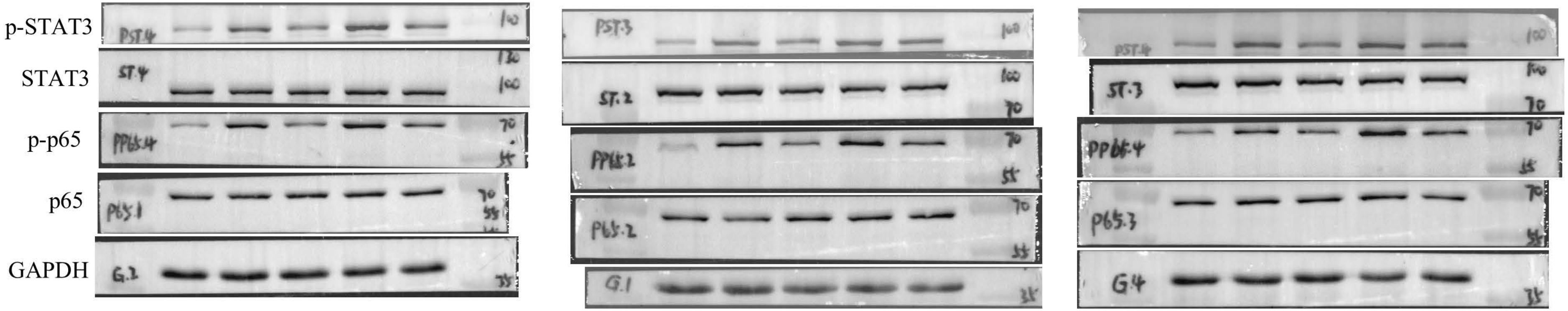


Figure 6G

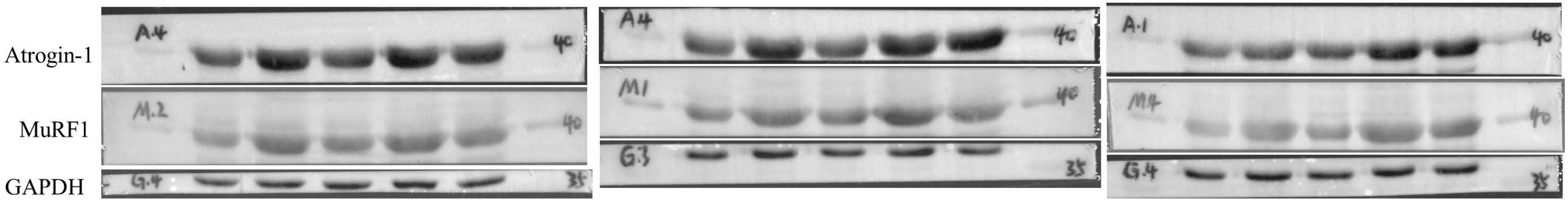

Figure 7A

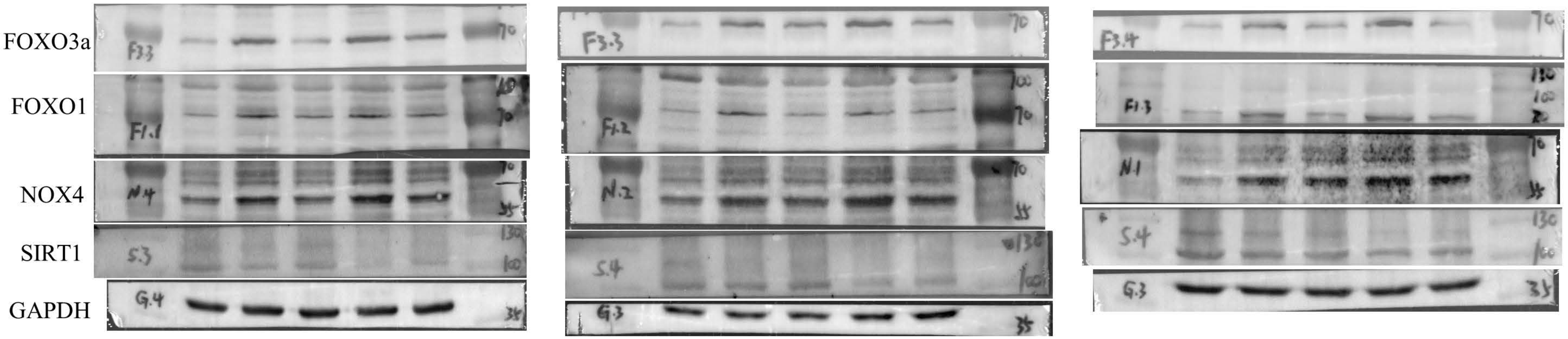


Figure 7B

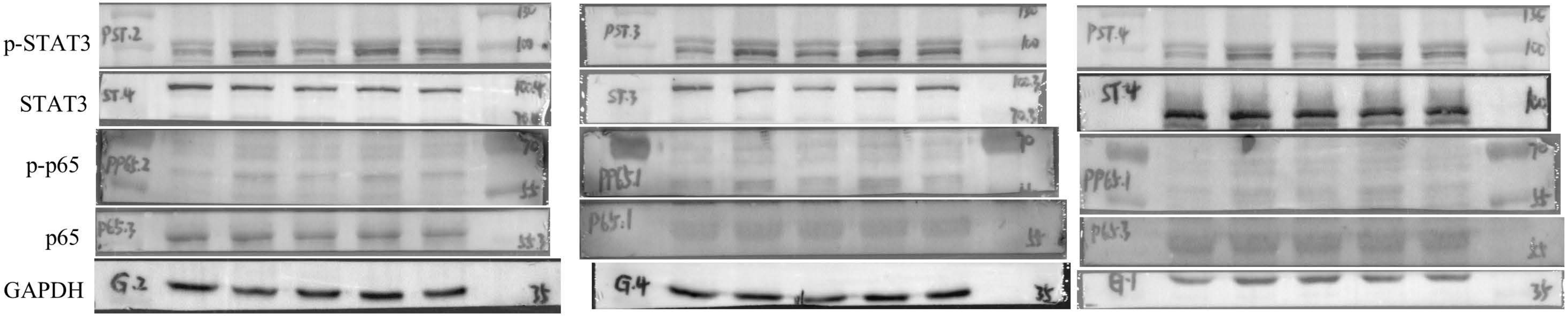

Supplement: Supplementary file 1 [file cancers-15-02378-s001.zip › cancers-2232080-original WB image.pdf]
