# Supplementary material for: Ursolic Acid Alleviates Cancer Cachexia and Prevents Muscle Wasting via Activating SIRT1
Source: Cancers (Basel). 2023 Apr 20;15(8):2378. doi: 10.3390/cancers15082378 (PMC10136986; doi:10.3390/cancers15082378)
Supplement: Supplementary file 1 [file cancers-15-02378-s001.zip › Supplementary material.pdf]

Ursolic acid alleviates cancer cachexia and prevents muscle wasting via activating SIRT1

Supplementary material

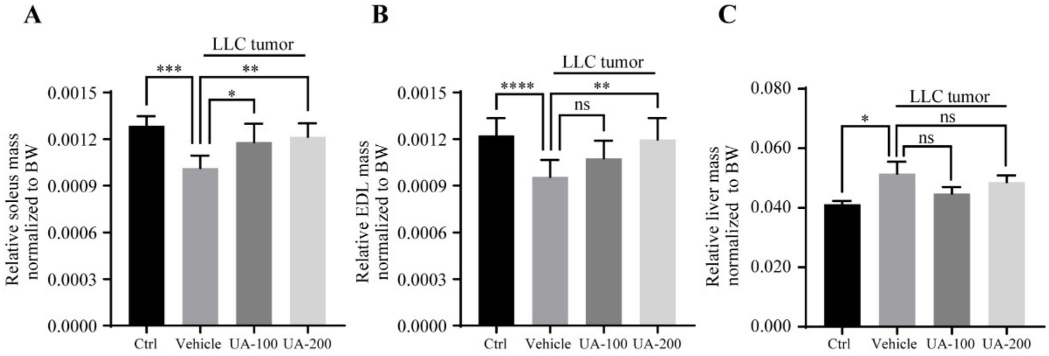

Figure S1. UA alleviates muscle wasting and prevents cancer cachexia in LLC tumor-bearing mice *in vivo*. The effects of UA on the **(A)** soleus mass, **(B)** EDL mass, and **(C)** liver mass of cachexia (BW: body weight), *$p < 0.05$, **$p < 0.01$, ***$p < 0.001$, ****$p < 0.0001$ versus control, ns: not significant, $n = 10$ mice/group.

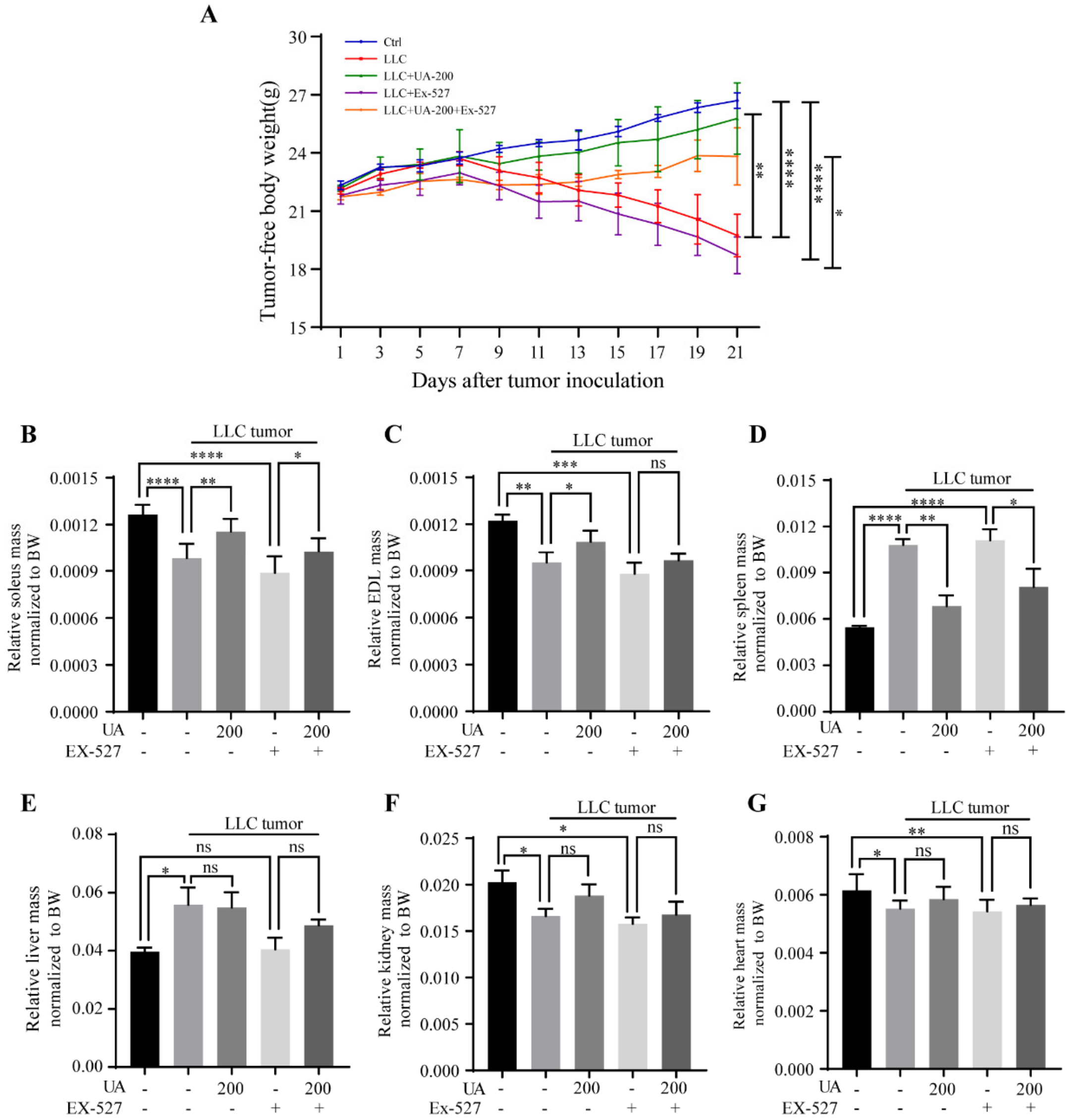

Figure S2. UA improves muscle wasting and prevents cancer cachexia in LLC tumor-bearing mice through SIRT1 activation. The effects of UA on the main features of cachexia were examined, including **(A)** tumor-free body weight, **(B)** soleus mass, **(C)** EDL mass, **(D)** spleen mass, **(E)** liver mass, **(F)** kidney mass, and **(G)** heart mass (BW: body weight), *$p < 0.05$, **$p < 0.01$, ***$p < 0.001$, ****$p < 0.0001$ versus control, ns: not significant, n = 10 mice/group.

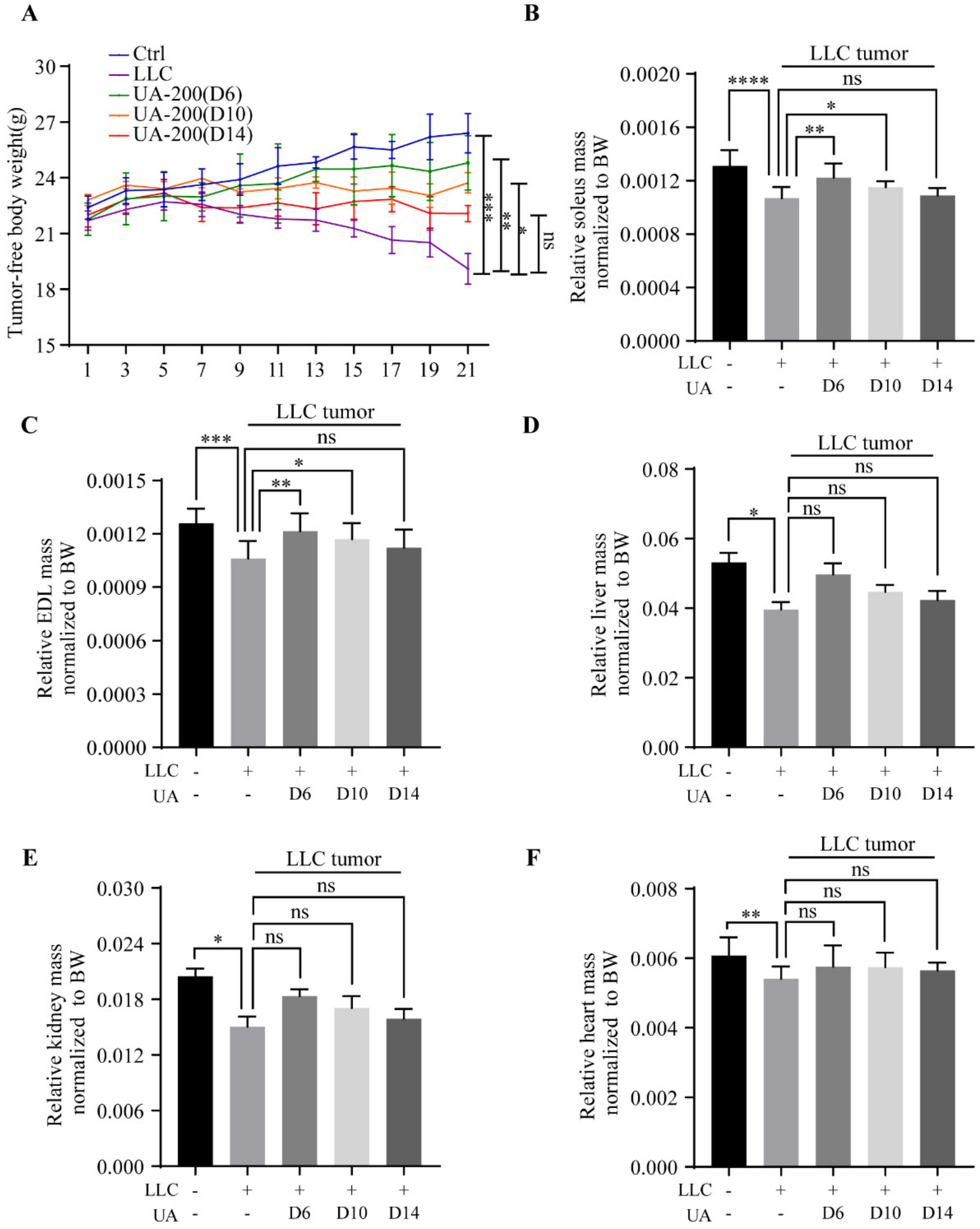

Figure S3. Effects of delaying treatment with UA until advanced stages of tumor and cachexia progression in LLC tumor-bearing mice. The effects of UA on the main features of cachexia were examined, including **(A)** tumor-free body weight, **(B)** soleus mass, **(C)** EDL mass, **(D)** liver mass, **(E)** kidney mass, and **(F)** heart mass (BW: body weight), $*p < 0.05$, $**p < 0.01$, $***p < 0.001$, $****p < 0.0001$ versus control, ns: not significant, $n = 10$ mice/group.
